# A Rare Case of Mania

**Published:** 1863-04

**Authors:** Hugh Grainger Stewart

**Affiliations:** Crichton Royal Institution.


### Art. V.—A RARE CASE OF MANIA.

By Hugh Grainger Stewart, M.D., Crichton Royal Institution.

It must be familiar to the observation of medical men having the charge of asylums for the insane, that a great deal can be done for the health, comfort, and tranquillity of the patients under their charge by careful attention to the regulation of the bodily functions of those entrusted to their care; that the quiet of a ward during the day, and the undisturbed rest of a maniac during the night, may be obtained by a regular, careful, and discriminating use of the most ordinary drugs in the Pharmacopœia. These are indeed important, but only minor results. It is also in the power of the physician to avert by the use of similar simple means very grave disease, to suspend the fury that would soon bring on a hopeless fatuity, and to give a fair chance for the operation of medicines whose mode of action is more obscure, and whose effects are less certain. These remarks apply more or less forcibly to cases of all varieties of mental disease, but their truth cannot be better exemplified than in epilepsy and chronic mania; especially in the former disease, a long series of the most violent fits may be frequently averted by the timely administration of a simple purgative. The following case exemplifies in a striking manner the benefit to be derived, in acute disease, from a careful observation of the condition of the bodily functions and the employment of simple measures to correct errors in them.

A gentleman, forty-five years of age, single, of independent means, but employed in an office, when sane of an obliging, kind, and cheerful disposition, of a good commercial education, and of quiet general deportment; has been for many years intemperate in his habits, and his excesses have given rise to frequent attacks of delirium tremens and to an impaired condition of health, which is supposed to be the cause of his insanity. For the last twenty years he has lived an active life and filled positions of responsibility creditably to himself and to the satisfaction of his employers. During this time he was in the habit of drinking a great deal, and when his duties were more than usually heavy, he used to stimulate his flagging energies with brandy during his working hours; his nights were spent in all kinds of debauchery, and it was often with difficulty that he could present himself to undertake the duties of the day. He found his health, which formerly was robust, under continued excesses and frequent attacks of delirium tremens, gradually give way. To check a further impairment of his health, he resolved at once to desist from all his evil habits; and accordingly eight years ago carried his purpose into effect, and has since abstained from all stimulants and other excesses. He remained well for three years, and prosecuted his duties at his office, but at the end of that period he became dull and desponding, looked back on his former life with intense sorrow, believed himself to be a very great sinner, dreaded eternal perdition, and sought comfort in reading the Bible and in telling his friends of his sins and short-comings. This change was the precursor of a severe attack of mania. Suddenly the patient became violent, disregarded all social obligations, looked on those whom he formerly regarded as friends as his bitterest enemies, and assaulted any one coming near him. He was furious and violent for several days, during which period he did not sleep; he then gradually recovered.

Similar attacks, however, occurred at varying intervals for the next three years, and were preceded, as the first had been, by a period of depression. During one whole year he was free from his malady, and was so well as to be able to return to his former duties. This immunity, however, was not permanent, for he was again prostrated, and in his violence nearly killed the woman in whose house he lodged, and against whom he entertained and cherished a murderous design; he also so publicly exhibited his malady, that it was thought necessary to seclude him, and he was accordingly placed under medical care. Having again recovered, he returned home, but symptoms appearing of another attack, he was placed in the Crichton Institution.

It was stated by his medical attendant that previous to his attack his liver became overloaded, his temper violent, and his conduct uncertain. He was also said to have suffered from epilepsy. No obvious change had taken place in the form of his insanity, but the recurring attacks were increasing in violence. His external senses were perfect, his appetite generally good, but his digestion and evacuations were disordered, whilst his pulse was regular, quiet, and of natural frequency, and his respiration tranquil. His attacks came on after intervals of from six weeks to three months. During the attacks the religious delusions above referred to were dominant, rendering his life miserable, and engrossing all his thoughts. During his lucid intervals he was free from delusion, and employed himself in walking and fishing, and, as has been before said, in resuming for a time at least his ordinary occupation. He has for the last eight years been regular in his habits and free from all excesses. It does not appear that there is or has been any hereditary disease or tendency to insanity in his family, but the means of information on this point are not so trustworthy as could be desired. He had been leeched and purged, and chloroform had been administered during the violent manifestations with advantage, and was under treatment in another asylum for four or five months.

The patient is of short stature, slight and active figure, has a large head in proportion to the size of the body, dark hair, and light blue restless eyes, congested eyeballs, and florid countenance, and is evidently nervous and excitable. On admission he was quiet and rational, but evidently depressed; he had his Bible or Prayer-book continually in his hand, and craved the ear and sympathy of his medical attendant, pouring forth a long history of his sins and shortcomings, and averring that he was only reaping what he had sown, now that these days of suffering had come on him. He slept ill the first night, and next day showed such symptoms of restlessness, that his attendant was cautioned to observe him carefully, and not without reason, for the anticipated attack came on shortly afterwards. With a cry he rushed at the man in charge of him, first trying to strike him down with a chair, and then grappling his throat in blind fury. He was placed in bed, and it required the constant attendance of two men, and sometimes more, to prevent him injuring himself and those about him. He continued furiously excited for four days, during which he slept none and ate little; only now and then, during a quiet interval, being induced to take a little water or some other cooling drink. There occurred during this period an immense number of attacks, in which the patient was furiously excited, blindly attacking his attendants in the fiercest manner, generally attempting to seize them by the throat and to bite their hands and arms, at the same time uttering the most terrific yells, and quivering in every limb. The eyes, apparently protruded, were turned rapidly from one side to another, as if anxiously looking for some vulnerable point of attack. The lips were retracted and the teeth displayed, ready to seize anything that might approach. Respiration was suspended until the face became livid. With a fearful yell a dash was made, every muscle in the body quivered and was exerted to its utmost; the awful struggle continued for some minutes, during which the countenance assumed the most appalling aspect, the lips and nostrils became almost black, and from the contorted face there burst forth the wildest and most awful laughter that ever human ear listened to. Again the patient would be quiet for a time, breathing would be resumed, and the sweat pour from him. But a few minutes at the most was the length of his respite; again he would begin to stare wildly about, and again would the fearful scene be repeated, the like of which had never before been witnessed either by the medical men or the most experienced attendants in the institution.

This continued day and night, without intermission, for three days; the attacks then became less frequent and severe, and gradually the patient resumed his ordinary condition, complaining of painful limbs and extreme exhaustion, the natural result of such extraordinary excitement.

Chloroform was administered copiously, and at first seemed to shorten the paroxysms, but in a short time appeared to lose its effect, and was abandoned. Sedatives were also administered, but with no benefit. The patient seemed to imagine himself surrounded by demons (he indeed stated so afterwards), and just at the outbreak of the attacks of fury, when talked kindly to, he appeared for a time to be able to control himself, but gradually the delusion overpowered him, and he gave way to his bursting passion. Purgative medicines were also administered, and with great benefit; the bowels had been overloaded, and the evacuations were dark in colour and very abundant.

For many weeks the patient was low in spirits and out of health after his severe affliction, but he gradually recovered, and became cheerful, active, and industrious. Occasionally, however, he was restless and depressed, complained of want of sleep, and mourned over his former course of life. He had, in fact, all the symptoms that on former occasions preceded his attacks. The treatment consisted of nourishing diet and the use of tonics and gentle aperients. His appetite was always good, and sometimes he ate enormously. It was observed that when his depressed state came on, a very active purge was necessary, and the accumulations in the primæ viæ were very abundant, fetid, and of a dark colour. These accumulations constantly took place, notwithstanding the administration of gentle purgatives, and the as regular occurrence of the alvine discharge; when removed, the patient became more cheerful, and the premonitory symptoms of more serious disease disappeared. Great attention was therefore paid to the condition of the bowels, and unless a copious discharge was produced, the inevitable precursors of the mania made their appearance. In addition to the use of a compound rhubarb pill every morning, at least once a week a strong dose of purgative medicine was administered, and was always followed by large evacuations. This treatment was persevered in for seven months with the effect of keeping the patient in very good health. He was always occupied, and rendered himself most useful; he was perfectly free from delusions and depressions, and looked forward with hope to the future.

About eight months after admission, he became dull, vigilant, and restless, and another attack was dreaded. On inquiry, it was found that the bowels had acted regularly, and that the other bodily functions were healthy. On percussing the abdomen, however, the left lumbar and iliac regions were found dull, and so distended as to lead to the conclusion that notwithstanding the use of medicine, an accumulation had taken place in the intestines. A strong drastic purgative was administered, but was not in time to avert the pending paroxysm. When present at a rehearsal for a concert of sacred music in which he was taking a part, and in assisting in which it was thought he might be enabled to withdraw his mind from his own morbid feelings, the patient suddenly uttered his fearful cry, and continued screaming and laughing alternately, offering no violence at first, apparently in mortal fear, and imploring God for mercy to pity him. There appeared to be no loss of consciousness, but the respiration was suppressed for a time and the respirations afterwards deep and long. On being placed in bed, he was in terror of immediate death; he was in a state of the greatest agitation, and cried out, "Oh, how shall I appear before the Great Judge?" "Have mercy on me!" and then came on a paroxysm of violence exactly similar to that which occurred eight months before. He had four such attacks during the night, but towards morning he was much better. There was no recurrence of the attacks; a little sleep was obtained, and the patient was calm and felt better. After the first attack, the medicine acted powerfully, and frequently afterwards during the night, the stools being large, dark-coloured, and of a most offensive odour. The treatment consisted in the administration of two drops of croton oil, which, however, were not retained by the stomach, and ten drops of the solution of atropine, ten drops containing the sixtieth of a grain of the drug, repeated in an hour; the result is already described. In a few days the patient was in his usual health, and now continues to enjoy life free from depression and anxiety. The rapid recovery from the last attack I entirely attribute to the purgative medicine; the atropine may have assisted in some way, but without the other would have been useless. Atropine has been observed in this institution to modify in a wonderful manner epileptic seizures, and for that reason was tried in the case under consideration, although not a case of epilepsy, yet having such a general resemblance to it in the mode of attack as to lead to a statement being made that the patient laboured under that disease.

The cry uttered at the commencement of an attack in this case was a cry for mercy or of fear, and the subsequent muscular movements were not convulsions, but were produced by terror, or arose from an instinct of self-preservation. There was no loss of consciousness, for impressions were received by the patient, and acted on as they were interpreted by him. He afterwards told me that at the moment of his crying out he believed he was suddenly called upon to appear before the Great

Judge, that a mighty terror came over him, and that he trembled and cried out. Afterwards he fancied himself surrounded by demons, and his efforts were for the purpose of freeing himself from them. At the commencement of a paroxysm it was possible to secure his attention for a moment, and an assurance that he was among his friends, and an appeal to his knowledge of those about him, for a moment delayed the attack. He saw and heard that such indeed was the truth; but the terror came over him again and overpowered his reason, and every impression received by the external senses was distorted by his mind into something that gave him additional proof of the reality of his supposed position. The opening and shutting of a door, a footfall in the adjoining gallery, the entrance of a stranger into his room, caused him immediately to start up, gaze wildly about him, cry out, and then struggle desperately. On afterwards endeavouring to recal his impressions, he could generally remember very accurately what had taken place; but he had forgotten some things—he had forgotten his own violence; he knew he had injured some of his friends, but when and in what manner he had no recollection. There would, therefore, appear to have been consciousness throughout these attacks, and the disappearance from the memory of certain events may be attributed to the dominance of other impressions.

The case was at first regarded as epileptic mania, partly because the notes accompanying the patient stated that he was subject to epilepsy, and partly from the description of his attacks, which resembled the ordinary manifestations of that disease. But the attacks described are evidently quite distinct from those occurring in epilepsy, and want the distinguishing characteristics of that malady. Consciousness was never lost, and the muscular movements were not convulsive—they were produced, indeed, by distinct acts of volition; there was no aura, or falling down; the respiration, although suppressed for a time, was evidently so for the purpose of giving greater power to the muscles, and the subsequent rapid breathing was the natural result of prolonged and violent muscular action.

The case may be called one of the *mania of Fear*. There were two periods in the attacks—the first, in which the patient was melancholy and uncertain in his conduct; and the second, in which mania was fully developed.

The question of causation is always an important one, and in this case especially so. The predisposing cause must lie in the constitution of the patient; his life of excess had produced this constitution of body to a certain extent. But a similar mode of life does not produce the same result in other individuals; we are therefore thrown back on anterior conditions, which, unfortunately, we have no means of knowing. In most cases, indeed, we are thus thrown out in investigating predisposing causes. The hereditary transmission of peculiarities from one individual to another comes in in the chain of causation; and then we must be satisfied, for it is only in rare cases that we can penetrate backwards in the history of individuals; and the sources of inheritance are so extensive in even two generations, that it is almost impossible to trace and distinguish them. The intemperance and other excesses in the case under consideration, however, are no doubt most important elements in the causation of the disease, but without the determining cause, now to be mentioned, would not have interfered very materially with the mental health of the individual. The exciting cause was apparently the accumulation of excrementitious matter in the intestines. When the patient first came under my observation there must have been torpidity of the bowels, for the purgatives given at that time brought away large quantities of fæces, and the patient soon recovered after getting rid of them. The history of the patient for seven months furnished almost a daily proof that the exciting cause was what is here mentioned, for on the slighest appearance of depression in the patient the remedy was administered, and the result was certain.

But perhaps the best proof afforded in the history of the case was the circumstances under which the last attack came on. These have been already narrated, and it was not until the primæ viæ were unloaded that symptoms of amendment showed themselves.

Since his last attack he has remained in his usual health, care being taken that medicine is administered in time to prevent any of the dreaded symptoms. The patient has a very good appetite, and is apt sometimes to overload his stomach, a tendency which in the treatment it is necessary to guard against. On these grounds I think it fair to draw the conclusion that the proximate cause of the maniacal attacks in this case depends on the accumulation of excrementitious matters in the intestines. How such a cause can produce such an effect, is another, a more difficult, and a question we will not attempt to solve. Whether the nervous system became poisoned by the presence of noxious matters in the blood, or whether irritation was produced in nerves which carried their impression to the brain, we cannot determine. Another question, important also, and especially so with regard to the radical cure, is, on what depends the torpidity of the intestinal canal? And this, too, we are unable to answer. There can, however, be no difficulty about the treatment; for constant observation and the administration of purgatives regularly to secure the thorough cleansing of the intestines, have procured for the patient very good health, both bodily and mental. By the use of simple medicines we are enabled to keep the patient in a sane state of mind, the incalculable value of which can only be appreciated by himself and by those who have seen him labouring under his fearful malady.

What has already and what is being done is a great deal; but can we not so improve the tone of the system of the patient that no external means will be necessary to ward off the disease? By the use of strychnia or some other medicines we may be able to give to the primæ viæ the power to perform effectually their functions, and thus produce a radical cure.

---

